# PLGA Nanofiber/PDMS Microporous Composite Membrane-Sandwiched Microchip for Drug Testing

**DOI:** 10.3390/mi11121054

**Published:** 2020-11-28

**Authors:** Wei Li, Xindi Sun, Bing Ji, Xingyuan Yang, Bingpu Zhou, Zhanjun Lu, Xinghua Gao

**Affiliations:** 1 Materials Genome Institute, Shanghai University, Shanghai 200444, China; liwei19981126@163.com (W.L.); sunxindi92@126.com (X.S.); yangxyshu@163.com (X.Y.); 2 Joint Key Laboratory of the Ministry of Education, Institute of Applied Physics and Materials Engineering, University of Macau, Taipa, Macau 999078, China; Jibing2015@hotmail.com (B.J.); bpzhou@um.edu.mo (B.Z.); 3 Department of Gastroenterology, Shanghai General Hospital, Shanghai Jiao Tong University School of Medicine, Shanghai 200080, China

**Keywords:** organ-on-a-chip, microfluidic chip, composite membrane, lung-on-a-chip, drug evaluation

## Abstract

Lung-on-a-chip devices could provide new strategies for a biomimetic lung cell microenvironment and construction of lung disease models in vitro, and are expected to greatly promote the development of drug evaluation, toxicological detection, and disease model building. In this study, we developed a novel poly (lactic-co-glycolic acid) (PLGA) nanofiber/polydimethylsiloxane (PDMS) microporous composite membrane-sandwiched lung-on-a-chip to perform anti-tumor drug testing. The composite membrane was characterized, and the results showed that it was permeable to molecules and thus could be used to study small-molecule drug diffusion. In addition, the microchip could apply perfusion fluids to simulate blood flow under extremely low fluid shear stress, and could also simulate the spherical-like shape of the alveoli by deformation of the composite membrane. Using this chip, we evaluated the anti-tumor drug efficacy of gefitinib in two kinds of non-small cell lung cancer cells, the lung adenocarcinoma NCI-H1650 cell line and the large cell lung cancer NCI-H460 cell line. We further probed the resistance of NCI-H460 cells to gefitinib under normoxic and hypoxic conditions. The established composite membrane-sandwiched lung chip can simulate more biochemical and biophysical factors in the lung physiological and pathological microenvironment, and it has important applications in the personalized treatment of lung tumors. It is expected to play a potential role in clinical diagnosis and drug screening.

## 1. Introduction

Microfluidic technology is a fluid manipulation technology that is widely used in basic sciences and interdisciplinary fields such as biology, materials, and chemistry, and shows significant application prospects in biomedicine and material synthesis [1,2,3,4,5,6]. With the continuous development of micromachining technology, especially the rapid advancement of soft lithography technology, organs-on-a-chip have attracted considerable attention [7,8,9]. Organs-on-a-chip, which are produced by combining microfluidic technology with micromachining and cell biology, are biomimetic systems capable of simulating the main functions of human organs. They have micron-sized cell culture chambers and fluid microchannels, which can simulate the main structural and functional characteristics of different tissues and organs of the human body, and thus the complex relationship among organs in vitro, to predict the body’s response to drugs or different external stimuli [8,9,10,11]. They have broad application prospects in the fields of life sciences and medical research, new drug development, personalized medicine, toxicity prediction, and individualized diagnosis [12,13,14].

In addition to microfluidic technology miniaturization, integration, and low consumption, organs-on-a-chip can precisely control biochemical or biophysical factors in cell microenvironments, such as chemical concentration gradients [15] and fluid shear stress [16]. It is also possible to construct cell patterns, tissue/organ interfaces, etc., to simulate the complex structure [17] and physiological functions of human organs [16]. In recent years, researchers have realized the construction of many human organs on microfluidic chips, such as lung-on-a-chip [18,19,20], liver-on-a-chip [21], gut-on-a-chip [22], kidney-on-a-chip [23], brain-on-a-chip [24], etc., to simulate pathologic conditions in physiologic cellular microenvironments, including pulmonary embolisms, pulmonary edema, and renal fibrosis, and to simulate intestinal flora and the construction of the blood–brain barrier.

The construction of these organs-on-a-chip is derived from the structural characteristics of organs or tissues to obtain specific shapes. The lung, for example, is a human respiratory organ whose basic unit is the pulmonary alveolus. There are hundreds of millions of pulmonary alveoli in the human body, which are the main places for gas exchange. They are space-filling cavities that have polygonal shapes with shared inter-alveolar walls, with an average diameter of about 200 μm. They include extremely rich biochemical/biophysical factors and are the only gas–liquid exchange interface in the human body: a gas exchange barrier layer with an average thickness of only a few microns, which is also called a respiratory membrane. The construction of a lung-on-a-chip is mainly carried out to simulate the respiratory membrane. The biological air–blood barrier can be considered a gas–liquid interface containing various factors [25,26,27,28,29].

Among the established lung-on-a-chip models, the earliest and most frequently used is a respirable lung-on-a-chip constructed by Huh et al. [30]. The chip uses a porous flexible polydimethylsiloxane (PDMS) membrane to divide the channel into an upper gas channel and a lower fluid channel, thus forming a gas–liquid interface and simulating the airflow of the airway and capillaries. The PDMS membrane also allows flexible deformation to simulate respiration. The lung chip model, developed based on microfluidic chip technology, is mostly a multilayer sandwich structure. The sandwich chip can simulate the trachea and blood vessels by using a microfluidic channel or simulate the gas flow rate and blood flow fluid shear stress by controlling the flow rate of gas or fluid [31]. Based on this sandwich structure, developing thin deformable membranes in microfluidic alveolar models from the group of O. Guenat has given us a new vision to explore how the chip uses mechanical stress in a dynamic nature and performs a wound healing assay. However, this kind of chip uses an electro-pneumatic set-up to control the contraction of membrane, which increases the difficulty in pulmonary research [32,33]. The membrane material in this sandwich structure can also be used to support a culture of relevant cells to simulate the respiratory membrane. Currently used membrane materials include porous flexible PDMS membranes, microporous silicon membranes, poly (lactic-co-glycolic acid) (PLGA) nanofiber membranes, etc., which have been used to conduct research on lung problems, including injuries, pulmonary embolisms, pulmonary inflammation, and tumors [34,35,36]. Because of the precise structure of the lungs and their complex factors, there is still room for improvement of the sandwich multilayer chip structure to simulate the lung environment, especially the membrane material itself.

**Figure 1:**
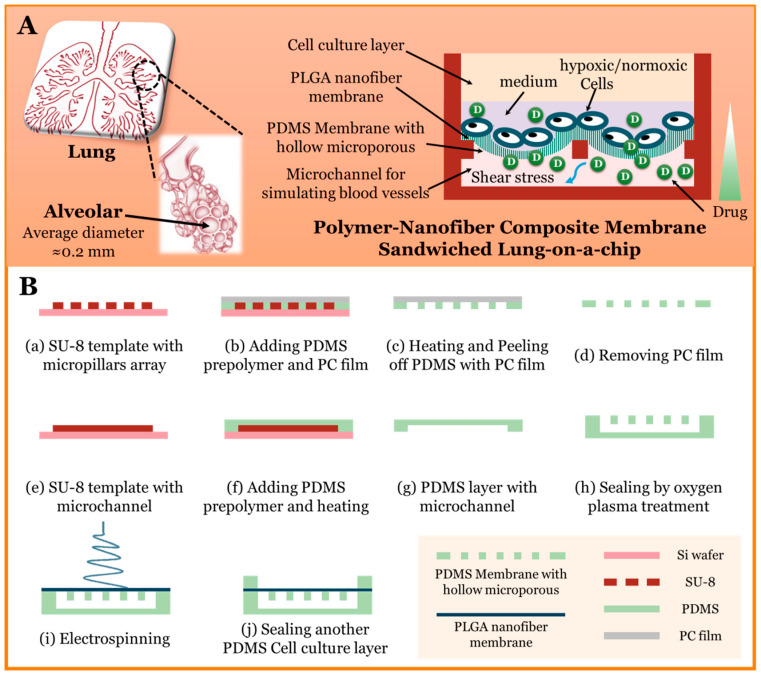
(**A**) Schematic diagram of chip design; (**B**) schematic diagram of microchip fabrication processes.

In our previous studies, we used a PLGA nanofiber membrane as the sandwich layer. However, PLGA nanofiber membrane was brittle and easily broken, which was not conducive to the fluid shear stress or terrain simulation [37]. In this study, we improved the preparation method and obtained a PLGA nanofiber/PDMS microporous composite membrane to develop a novel lung-on-a-chip, a schematic diagram which is shown in Figure 1A. The microchip simulated both blood flow under extremely low fluid shear stress and the shape of the alveoli. We also used this microchip to study the sensitivity of lung cancer cells to the targeted drug gefitinib under normoxic and hypoxic conditions. The drug testing in our research is done using the EGFR inhibitor gefitinib, which is a first-line therapy against the EGFR-mutated non-small cell lung cancer, followed by detecting the expression of epidermal growth factor receptor (EGFR) under normoxic condition and hypoxia-inducible factor 1-alpha (HIF-1α) under hypoxic condition. EGFR has been the most comprehensively studied molecular target and has shown highly promising activity in the clinical oncology therapeutics over the past decade [38].

## 2. Materials and Methods

### 2.1. Microchip Fabrication and Characterization

In this study, the microchip was multilayered and included a PDMS microchannel perfusion layer with six parallel units for simulating blood vessels, a sandwiched layer of PLGA nanofiber/PDMS microporous composite membrane, and a PDMS cell culture layer. The fabrication processes are shown in Figure 1B. We first prepared the PDMS microporous membrane by the following method. We prepared a SU-8 (3035, MicroChem Corp., Westborough, MI, USA) template with a micropillar array by soft lithography and then added the prepolymer PDMS (Sylgard 184, Dow Corning Corp., Auburn, AL, USA, curing agent: monomer = 1:10) to the template, covered it with a piece of polycarbonate (PC) film (3M), pressed the PC film down with a heavy object (~1 Kg), and then heated it to polymerize the PDMS at 80 °C for 60 min. In order to avoid bubbles, we used a vacuum oven to remove bubbles in PDMS prepolymer after it was added to the template. The PC film was then removed to obtain a PDMS microporous membrane. We also used soft lithography to prepare the microchannel perfusion layer and then treated the microchannel perfusion layer and microporous membrane with oxygen plasma for bonding. Finally, electrospinning was performed on the side of microporous membrane to obtain a PLGA nanofiber membrane. The preparation method of PLGA nanofiber membrane was based on [37]. The PDMS cell culture layer and other layers were fixed with a clamp to ensure that the cell culture chamber was not leaking. The PDMS cell culture layer has six open cell culture chambers, so that the microfluidic device was ever exposed to air or under a hypoxic condition. We used a Surface Profiler (Alpha-step D-300 stylus profiler, KLA Tencor Corp., Milpitas, CA, USA) to measure the thickness of the PLGA nanofiber membrane. We also characterized the PLGA nanofiber/PDMS microporous composite membrane using a scanning electron microscope (SEM, Hitachi High-technologies Corp., Tokyo, Japan) and an inverted microscope (IX 73, Olympus Corp., Tokyo, Japan).

### 2.2. Fluid Perfusion and Computational Fluid Dynamics Simulation

**Figure 2:**
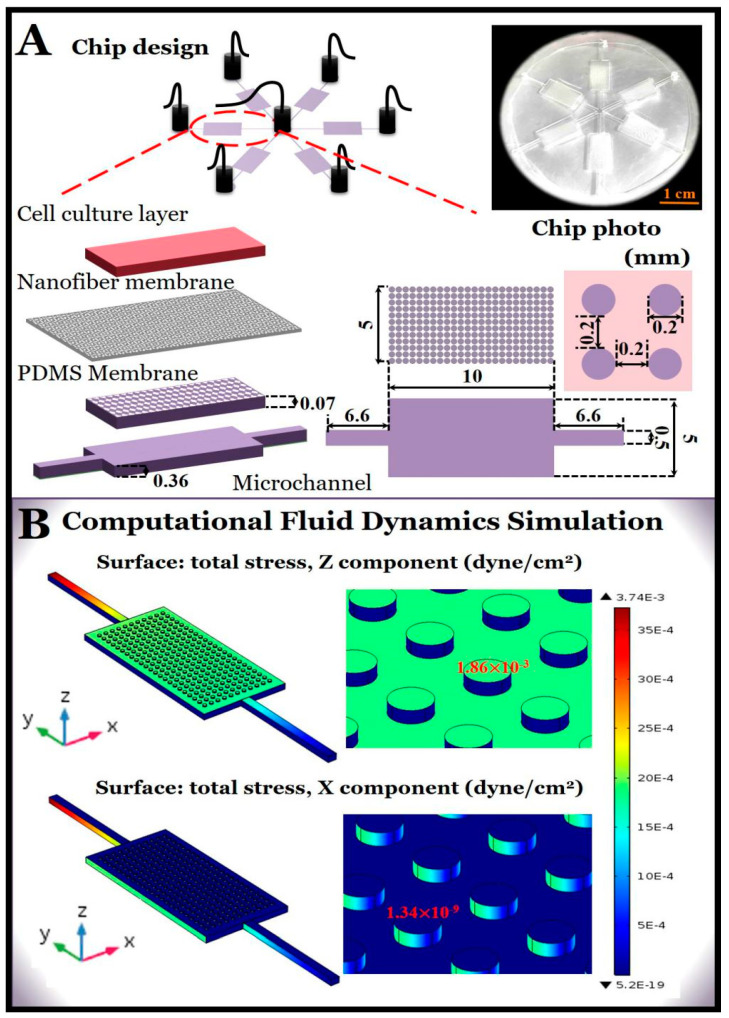
(**A**) Structure and size of multi-layered lung chips. The inset shows a photograph of the chip. The shot is taken from the angle of the microchannel layer and shows the microchannel of the chip and the white poly (lactic-co-glycolic acid) (PLGA) fiber membrane; (**B**) the results of computational fluid dynamics simulation. The shear stresses in the Z and X components were located on the surface of the composite membrane in the perfusion microchannel.

To simulate the slow blood flow within capillaries, we performed fluid perfusion on the microchip using a syringe pump (Harvard Apparatus, Holliston, MA, USA). The established microchip had six parallel units, with six liquid inlets on the outside and one liquid outlet in the middle. For one unit, the perfusion rate was 1 mL/h in experiments requiring perfusion. To better describe the perfusion condition, we used computational fluid dynamics software COMSOL (Multiphysics v4.4, COMSOL Inc., Burlington, MA, USA) to simulate the fluid shear stress of one of the units. The specific dimensions of the chip are shown in Figure 2A. The microchannel was about 360 µm high, and the PDMS microporous array was a 200 µm diameter hollow hole with a hole spacing of 200 µm, a thickness of about 70 µm, and a PLGA nanofiber membrane thickness of about 3 µm. A photograph of the multilayer chip is shown in the inset of Figure 2A.

### 2.3. Drug Diffusion and Detection

To characterize the diffusion of the PLGA nanofibers/PDMS microporous composite membranes for small-molecule drugs, we used rhodamine 123 (Rh-123, Sigma, St. Louis, MO, USA) as the dye for the diffusion experiments. We configured an Rh-123 aqueous solution with a final concentration of 1 μg/mL (≈2.63 µM), which was used as a perfusion liquid, with 1 mL/h applied to one unit of the microchip. Then, 500 μL of pure water was added to the cell culture layer of the unit, which was mixed with a pipette every few hours; 2 μL were taken for the microplate reader (Multiskan GO, Thermo Fisher, Waltham, MA, USA). The concentrations of Rh-123 in the cell culture layer were roughly calculated by ultraviolet (UV)-visible spectrophotometry. The standard curve is shown in Supplementary 1.

### 2.4. Cell Culture and Seeding

Two human non-small cell lung cancer lines were used. One was the lung adenocarcinoma NCI-H1650 cell line and the other was the large cell lung cancer NCI-H460 cell line. Both were obtained from the Cell Bank/Stem Cell Bank of the Chinese Academy of Sciences (source: ATCC) and cultured in Roswell Park Memorial Institute Medium 1640 (1640, Gibco, Grand Island, NE, USA) containing 10% fetal bovine serum (FBS, Gibco) and 1% penicillin-streptomycin antibody (Gibco). For NCI-H1650 or NCI-H460 cell cultures in the microchip, 1 mL cell suspension with a density of 2 × 10^5^ /mL was added to each unit in the cell culture layer. After 24 h, the cells were attached to the PLGA nanofibers for subsequent operations.

### 2.5. Cell Staining and Imaging

Immunofluorescence staining was used to detect the expression of epidermal growth factor receptor (EGFR) and hypoxia-inducible factor 1-alpha (HIF-1α). A standard immunofluorescence staining process was performed. Primary antibodies of rabbit anti-human EGFR and rabbit anti-human HIF-1α, a secondary antibody of goat anti-rabbit IgG marked by Alexa Fluor 568 or Alexa Fluor 488, and a blocking buffer were purchased from Abcam. The 4′,6-diamidino-2-phenylindole (DAPI) for cell nucleus staining was purchased from Sigma. The images were taken by an inverted fluorescence microscope (IX 73, Olympus).

### 2.6. Drug Treatment and Cell Viability Test

In this experiment, we selected the EGFR-targeted drug gefitinib (Sigma) for drug evaluation. A serum-free medium with a final concentration of 2.2 µM gefitinib was prepared and applied to the cells in different ways, including either directly into the cell culture layer or through perfusion flow layer. To study the cellular drug susceptibility under normoxia and hypoxia conditions, we also applied different oxygen concentrations (21% normoxia and 1% hypoxia) using a cell culture CO_2_ incubator (Thermo Fisher Scientific). After cell treatment for 24 h, the cell viability was detected by calcein-AM (Life Invitrogen, Carlsbad, CA, USA) staining and photographed using a laser scanning confocal microscope (FV 3000, Olympus).

### 2.7. Data Statistics

Image J software or confocal microscope software was used for the data statistics and analysis of the cell images and videos, mainly including the average relative fluorescence intensity of cells and the results of cell slide scanning in the Z direction. The standard errors in the experimental data were obtained from Student′s *t*-test under multiple groups of data (*n* ≥ 3). 

## 3. Results and Discussion

### 3.1. Chip Design and Flow Simulation

In a normal physiological structure, the surfaces of the alveoli are surrounded by capillaries and the blood flow is usually slow (~0.1–60 dyne/cm^2^ [39]). In this study, we simulated the slow movement of fluid in the capillaries under a condition of perfusion. To determine whether the flow rate in the microchip was appropriate, we first used the COMSOL software to perform a computational fluid dynamics simulation. The COMSOL model was established in a three-dimensional structure with the same size as experiments. The laminar single-phase flow was selected to track the shear stress and the governing equations in the model were the Navier–Stokes Equation (1) and the continuity Equation (2)

(1) ρ ∂ν/∂t+ρ(ν⋅∇)ν=−∇p+μ∇^2 ν,

(2) ∇⋅ν=0,

where *ρ* and *μ* are the density and dynamic viscosity of the fluid, respectively, ***υ*** is the fluid velocity vector, and *p* is the pressure. In this model, a typical flow rate of 1 mL/h was set in the inlet under the laminar inflow condition, and the density and dynamic viscosity of the fluid were set as 1 × 10^3^ kg/cm^3^ and 1 × 10^−3^ Pa·s, respectively. From the simulation results (Figure 2B), it can be found that the fluid shear stress at X component has restricted influence on the top microporous membrane, which imitates the alveoli, and is therefore negligible, which is also applicable to the one at Y component. The shear stress at Z component, however, was approximately 10^−3^ dyne/cm^2^, which is in the range of fluid shear stresses in the interstitial fluid flow in human body. Usually the fluid shear stress of the interstitial flow is within the range from ~10^−2^ to 10^−4^ dyne/cm^2^ [39]. Thus, in later experiments, we used a perfusion flow rate of 1 mL/h to simulate blood flow in capillaries with very low fluid shear stress.

### 3.2. Characterization of Composite Membrane

**Figure 3:**
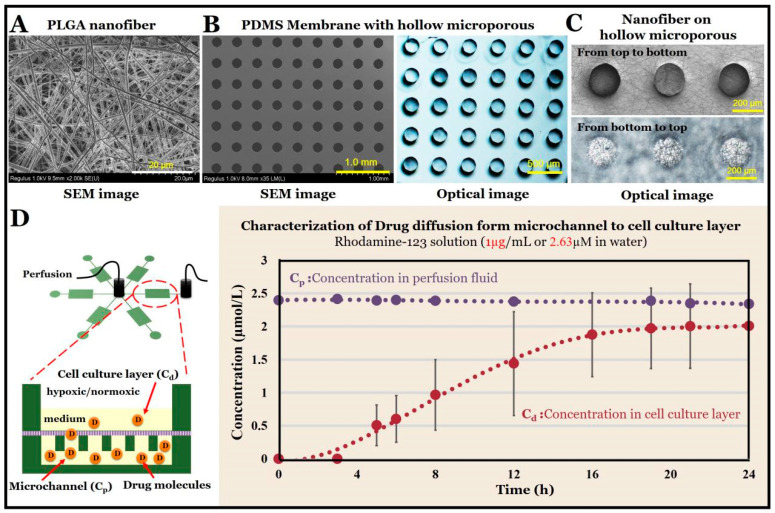
(**A**) Scanning electron microscope (SEM) image of PLGA nanofibers. (**B**) SEM and optical images of the polydimethylsiloxane (PDMS) hollow microporous membrane. (**C**) Optical images of the composite membrane in different directions. (**D**) Characterization of drug diffusion from the microchannel to the cell culture layer. A 2.63 µM Rhodamine-123 solution in water was perfused into the microchannel at 1 mL/h. The cell culture layer was initially filled with 500 μL of pure water; after perfusion, the absorbance was detected every several hours. (*n* = 5) The drug concentration in the cell culture room and in the perfusion fluid were determined by standard UV-visible spectrophotometry.

We characterized the PLGA nanofiber/PDMS microporous composite membrane by SEM and optical microscopy. Figure 3A shows an SEM image of the PLGA nanofibers, Figure 3B shows SEM and optical images of the microporous PDMS membrane, and Figure 3C shows optical images of the PLGA nanofiber/PDMS microporous membrane. The PLGA spinning solution we used in the experiment was 10% PLGA in 2,2,2-trifluoroethanol. It can be observed that the PLGA nanofibers have a smooth surface and uniform size, forming a porous network. The PLGA nanofiber membrane was measured to be about 3 µm thick using a surface profiler. The microporous PDMS membrane was about 70 μm thick, and the micropores were 200 μm in diameter and regularly spaced 200 μm apart. In addition, it can be observed that in the composite membrane obtained in the experiment, the microporous array of the PDMS membrane were well covered with PLGA nanofibers.

Because the composite membrane we prepared had hollow micropores and the network formed by the PLGA nanofibers was also porous, we believe that the composite membrane would allow small molecules to pass through. This has also been confirmed in previous work [37]. Thus, we used Rh-123, which has a molecular weight close to that of gefitinib, as a dye for the drug to verify drug diffusion. We added Rh-123 to the perfusion flow and a blank solution to the cell culture layer and measured the absorbance of both solutions every few hours. The Rh-123 concentration was calculated by the standard curve, and the results are shown in Figure 3D. The Rh-123 concentration in the perfusion flow did not change much, but the concentration in the cell culture layer gradually increased with time, indicating that small molecules passed through the pores of the composite membrane into the cell culture layer solution.

### 3.3. Cellular Activity and Membrane Deformation

**Figure 4:**
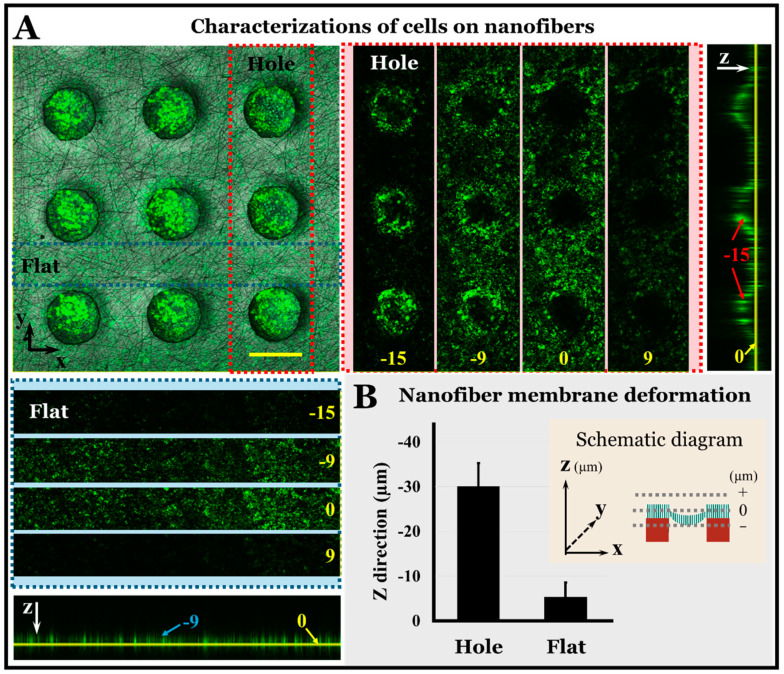
(**A**) Fluorescent images of cells on composite membrane by laser scanning confocal microscope. The plan view in the XY direction is a superposition of fluorescence and bright images at 0 µm in the Z direction. The horizontal illustrations are fluorescence images of cells on the fiber surfaces of hollow micropores in the direction of the Z axis layer by layer. The longitudinal illustrations are fluorescence images of cells on the fiber surfaces of flat PDMS membranes in the direction of the Z axis layer by layer. Cell: green. Bar = 200 µm. (**B**) Histogram of nanofiber membrane deformation in Z-direction. *n* = 9.

We detected the adhesion of NCI-H460 cells on the composite membrane of the established lung chip. We added 1 mL of cell suspension directly to the cell culture layer and cultured it for 24 h without applying the perfusion flow. After cell adhesion, NCI-H460 cells were stained by calcein-AM, and the results showed that the cells survived well on the surfaces of the PLGA fibers. In addition, we found that the PLGA nanofibers on the hollow microporous PDMS membrane were bent and deformed owing to the static pressure of the solution. Thus, we used a laser confocal scanning microscope to perform a slice scan in the Z direction every 3 μm. The results are shown in Figure 4 and the scan video in the Z-direction is shown in Supplementary 2. In this case, we used the cell position to characterize the deformation of the membrane. We set the 0 µm position to be the upper surface of the PLGA nanofibers, and the PLGA nanofibers were about 3 µm high. It can be observed that when the cells were on the fiber surfaces of the flat PDMS, the cells appeared at −5.3 ± 3.3 µm in the Z direction. However, when the cells were on the fiber surface of the hollow microporous membrane, the cells appeared at −30.0 ± 5.20 µm in the Z direction. Although the cells were in a static culture mode, the deformation of the composite membrane seem similar to a spherical shape, which makes the cell layer similar to the shape of alveoli during breathing. This cellular activity and membrane deformation show the air-blood barrier and the air-liquid interface in the lung-on-a-chip design, which simulate more biochemical and biophysical factors of the physiological and pathological microenvironment in the lung, and it has important applications in the personalized treatment of lung tumors. It is expected to play a potential role in clinical diagnosis and drug screening.

### 3.4. EGFR-Targeted Drug Evaluation under Normoxia Conditions

**Figure 5:**
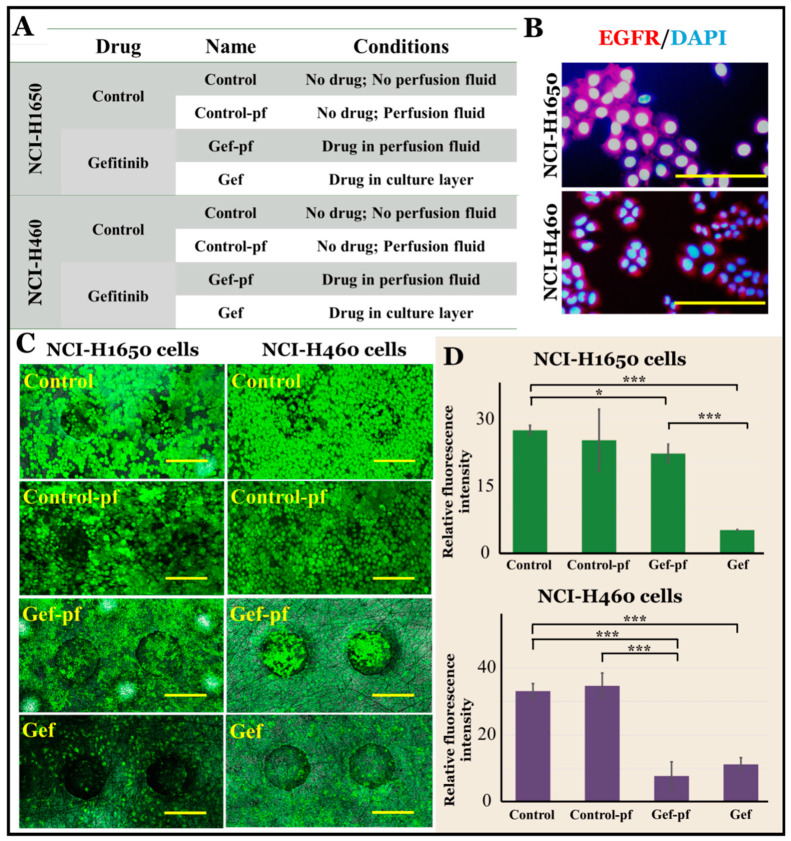
(**A**) Grouping and labeling of NCI-H1650 cells and NCI-H460 cells drug evaluations under normoxic condition. (**B**) EGFR expression of NCI-H1650 cells and NCI-H460 cells. EGFR: red; DAPI for cell nucleus: blue. Bar = 100 µm. (**C**) Calcein-AM fluorescence image of NCI-H1650 cells and NCI-H460 cells in different groups under normoxic condition. calcein-AM: green. Bar = 200 µm. (**D**) Histogram of the average relative fluorescence intensity of calcein-AM staining of NCI-H1650 cells and NCI-H460 cells in different groups under normoxic condition. The standard errors were obtained from Student’s *t*-test. *n* = 4; *** *p* < 0.005; * *p* < 0.05.

On the established lung chip, we carried out drug evaluations on lung adenocarcinoma NCI-H1650 cells and large cell carcinoma cells NCI-H460 cells under normoxia conditions. Different groups were used to consider the effects of drug diffusion under perfusion, including a control group without the drug (Control), another control group without the drug in perfusion flow (Control-pf), a drug group with 2.2 µM gefitinib in perfusion flow (Gef-fp), and a drug group with 2.2 µM gefitinib in a cell culture layer (Gef). The group and tag details are shown in Figure 5A. As gefitinib is an EGFR-targeted anti-tumor drug, we also carried out immunofluorescence staining of EGFR expression on NCI-H1650 cells and NCI-H460 cells, as shown in Figure 5B. Both cell types were bright red after immunofluorescence staining, and they were EGFR-positive cells.

We used calcein-AM staining to identify cell viability, and the results are shown in Figure 5C,D. For both NCI-H1650 cells and NCI-H460 cells, it can be observed that there is not much difference between Control group and Control-pf group, indicating that perfusion flow has no effect on cell activity. After 24 h of drug treatment, the cell-staining results of the Gef-fp and Gef groups changed significantly compared with the control group; that is, the fluorescence intensity decreased. In addition, for the NCI-H1650 cells, we found that the Gef-pf group cells had higher activity than the Gef group. The probable reason is that in the Gef-pf group, the estimated maximum concentration of drug diffusing into the cell culture layer was lower than that of the drug directly applied to the cells in the cell culture layer (Gef group). Because the drug diffusion required more time, which resulted in a low concentration and short time of drug treatment for cells in the Gef-pf group, the anti-tumor drug gefitinib had a weakened effect, and the tumor cell activity was better. However, under the same conditions for the NCI-H460 cells, whether in the Gef-pf group or the Gef group, the cell activities were similar and low, indicating that the NCI-H460 cells were more sensitive to gefitinib, and the concentration of gefitinib after diffusion was sufficient to kill tumor cells.

### 3.5. EGFR-Targeted Drug Evaluation under Hypoxia Conditions

**Figure 6:**
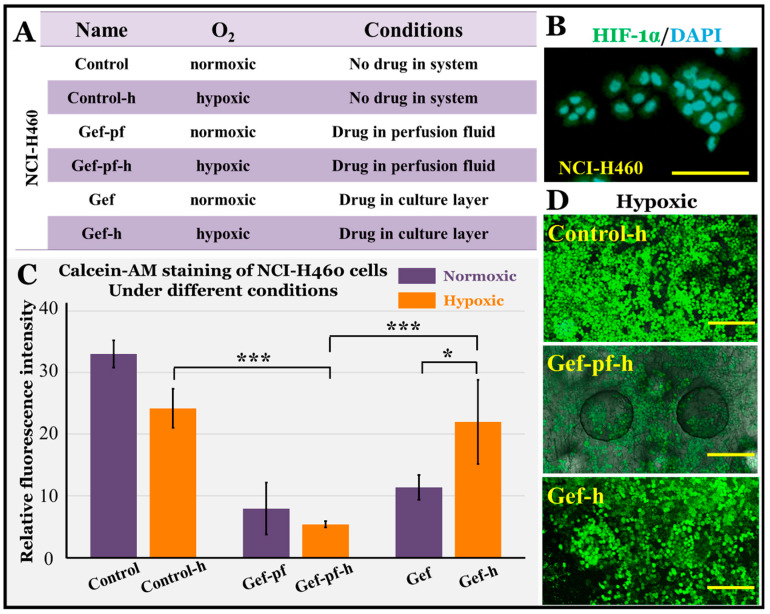
(**A**) Grouping and labeling of NCI-H460 cells drug evaluation under normoxic and hypoxic conditions. (**B**) HIF-1α expression of NCI-H460 cells. HIF-1α: green; DAPI for cell nucleus: blue. Bar = 100 µm. (**C**) Histogram of the average relative fluorescence intensity of calcein-AM staining of NCI-H460 cells in different groups under normoxic and hypoxic condition. The standard errors were obtained from Student’s *t*-test. *n* = 4; *** *p* < 0.005; * *p* < 0.05. (**D**) Calcein-AM fluorescence image of NCI-H460 cells in different groups under hypoxic condition. Calcein-AM: green. Bar = 200 µm.

Usually, patients with lung cancer have symptoms such as cough, chest tightness, inflammation, etc., and a certain degree of poor breathing and tissue hypoxia. The interior of solid tumors is often in a state of greater hypoxia, which further simulates the pathological condition of the lungs [39,40]. Therefore, in addition to the above normoxic conditions, we also carried out drug evaluation under hypoxic conditions (oxygen concentration 1%) by providing an oxygen-deficient atmosphere in the incubator. The group and tag details are shown in Figure 6A. To verify the hypoxia of the cells, we cultured NCI-H460 cells with better drug sensitivity for 48 h under hypoxic conditions and applied immunofluorescence staining for HIF-1α. HIF-1α is a marker of the degree of hypoxia in cells and tissues and is associated with cell survival, proliferation, and drug resistance. The results are shown in Figure 6B. It can be observed that the expressions of HIF-1α were positive, demonstrating that the cells were indeed under hypoxic conditions.

We then identified the NCI-H460 cell viability by calcein-AM staining. The results are shown in Figure 6C,D. For ease of comparison, the NCI-H460 cell viability data under normoxic conditions are shown again in 6C. The conditions for the different groups under hypoxic conditions were similar to those under normoxic conditions, including the control-h group without the drug, the Gef-h group with 2.2 µM gefitinib injected directly into the cell culture layer, and the Gef-pf-h group with 2.2 µM gefitinib in perfusion flow. We observed that the cell viabilities of the control-h and Gef-h groups were better. Among them, the Gef-h group was significantly different from the normoxic Gef group in that that the cells had gefitinib resistance. These results are similar to those of some working reports, which found that hypoxia induced gefitinib resistance in non-small cell lung cancer, both mutant and wild-type [41]. In the Gef-pf-h group, however, the NCI-H460 cells were still sensitive to gefitinib. There was a significant difference in cell viability between the Gef-pf-h group and the control-h/Gef-h groups. We speculated that this might be due to the fact that perfusion fluid was a continuous fresh medium that carried a certain amount of oxygen, so there was no hypoxia. There was no significant difference between the cell activity of the Gef-pf-h group and that of the Gef-pf group. It can also be seen that in the pathological condition of the body, the oxygen supply can improve after the tumor induces neovascularization. At this time, because of the appearance of new blood vessels, the tumor may already be in the middle and late stages, and its drug resistance may be a more complicated, multi-factor problem, which requires more research in future.

## 4. Conclusions

In this study, a PLGA nanofiber/PDMS microporous composite membrane-sandwiched lung-on-a-chip microdevice was developed. The composite membrane was permeable to molecules and was used to study small-molecule drug diffusion. Perfusion fluid could be applied to the established lung chip to simulate blood flow under extremely low fluid shear stress, and the chip simulated the spherical-like shape of the alveoli by deformation of the composite membrane. Normoxia and hypoxia atmospheres were also successfully applied. We carried out gefitinib drug testing for lung non-small cell lung cancer and investigated cell activity by calcein-AM staining. The activity of HCI-H1650 cells under normoxic conditions was correlated with the concentration and timing of gefitinib. However, NCI-H460 cells were more sensitive to gefitinib and exhibited certain resistance under hypoxic conditions. Besides, this composite membrane is applicable that normal and healthy or primary epithelial cells can also be used to replace lung tumor cells for other tests. The established composite membrane sandwich lung chip effectively solves problems that occur when using PLGA nanofiber membranes alone. It simulates more biochemical and biophysical factors of the physiological and pathological microenvironment in the lung, and it has important applications in the personalized treatment of lung tumors. It is expected to play a potential role in clinical diagnosis and drug screening.

## Data Availability

The data that support the findings of this study are available from the corresponding author upon a reasonable request.

## Supplementary Materials

The following are available online at https://www.mdpi.com/2072-666X/11/12/1054/s1. Supplementary 1. Standard curve obtained by UV-Vis spectrophotometry for Rh-123. Supplementary 2. Z-direction slice scanning video from laser confocal microscope, cell: green.
