# Supplementary figures and images for: PLGA Nanofiber/PDMS Microporous Composite Membrane-Sandwiched Microchip for Drug Testing
Source: Micromachines (Basel). 2020 Nov 28;11(12):1054. doi: 10.3390/mi11121054 (PMC7760955; doi:10.3390/mi11121054)

## Supplementary 1

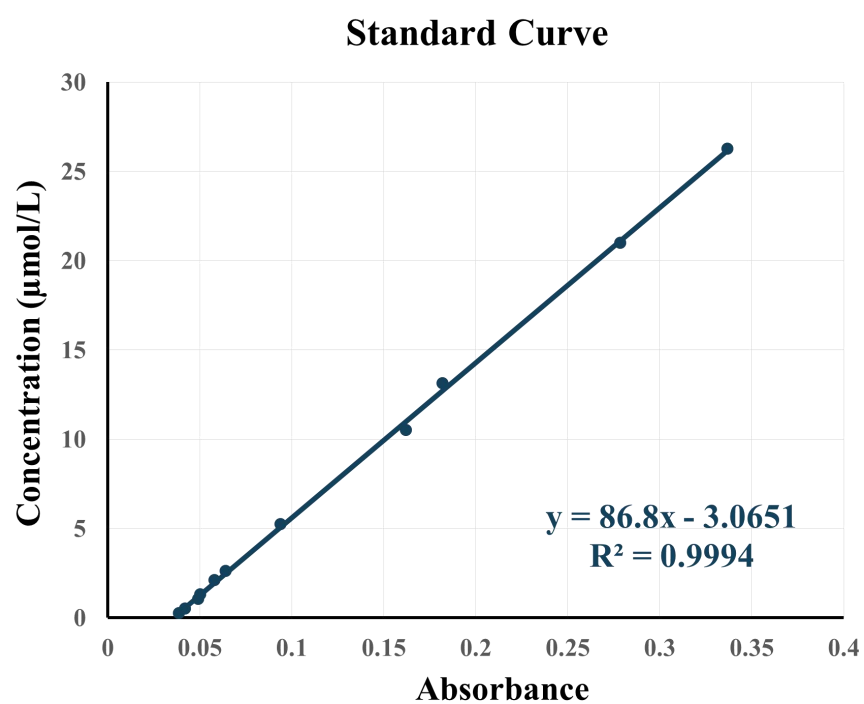

Supplement: Supplementary file 1 [file micromachines-11-01054-s001.zip › Supplementary Materials/Supplementary 1.pdf]
